# Toward Structural Health Monitoring with the MyShake Smartphone Network

**DOI:** 10.3390/s23218668

**Published:** 2023-10-24

**Authors:** Sarina C. Patel, Selim Günay, Savvas Marcou, Yuancong Gou, Utpal Kumar, Richard M. Allen

**Affiliations:** 1 UC Berkeley Seismology Lab, Berkeley, CA 94720, USA; savvas.marcou@berkeley.edu (S.M.); yuancong_gou@berkeley.edu (Y.G.); utpalkumar@berkeley.edu (U.K.); rallen@berkeley.edu (R.M.A.); 2 Pacific Earthquake Engineering Research Center, Berkeley, CA 94720, USA; selimgunay@berkeley.edu

**Keywords:** smartphones, structural health monitoring, fundamental frequency, MyShake, seismology, shake table

## Abstract

The field of structural health monitoring (SHM) faces a fundamental challenge related to accessibility. While analytical and empirical models and laboratory tests can provide engineers with an estimate of a structure’s expected behavior under various loads, measurements of actual buildings require the installation and maintenance of sensors to collect observations. This is costly in terms of power and resources. MyShake, the free seismology smartphone app, aims to advance SHM by leveraging the presence of accelerometers in all smartphones and the wide usage of smartphones globally. MyShake records acceleration waveforms during earthquakes. Because phones are most typically located in buildings, a waveform recorded by MyShake contains response information from the structure in which the phone is located. This represents a free, potentially ubiquitous method of conducting critical structural measurements. In this work, we present preliminary findings that demonstrate the efficacy of smartphones for extracting the fundamental frequency of buildings, benchmarked against traditional accelerometers in a shake table test. Additionally, we present seven proof-of-concept examples of data collected by anonymous and privately owned smartphones running the MyShake app in real buildings, and assess the fundamental frequencies we measure. In all cases, the measured fundamental frequency is found to be reasonable and within an expected range in comparison with several commonly used empirical equations. For one irregularly shaped building, three separate measurements made over the course of four months fall within 7% of each other, validating the accuracy of MyShake measurements and illustrating how repeat observations can improve the robustness of the structural health catalog we aim to build.

## 1. Introduction

Structural health monitoring (SHM) performed via the vibration response of built structures is a well-developed field [1,2,3]. Nonetheless, the ’barrier to entry’ associated with the instrumentation of buildings and infrastructure presents a significant challenge to actually monitoring structures around the world. Traditional monitoring projects require permits, the installation of specialized sensors, and ongoing maintenance, all of which are costly in terms of time, human power, and resources [4]. Consequently, only a minuscule fraction of all buildings is actually instrumented. For example, there are only ∼340 instrumented buildings in California for which records are available in the Center for Engineering Strong Motion Data (CESMD) database [5].

The use of personal smartphones as vibrational sensors represents a novel solution [6,7,8] as smartphone ownership is becoming increasingly globally ubiquitous [9]. With an estimated 6.6 billion smartphone subscriptions globally in 2022 [10], smartphones are increasingly likely to be found in most buildings, particularly in urban areas. Previous work evaluating smartphones for SHM has used dedicated phones fixed in specific locations [8,11], leveraged the phone camera for visual damage inspection [12], used the onboard GPS to detect large ground motions [13], enabled individual researchers to view vibrational frequency content [7,14,15], or experimentally estimated smartphone performance using a mechanical shaking apparatus [8,16,17,18]. The MyShake project seeks to capitalize on the ubiquity of personal smartphones by employing their in-built accelerometers to crowd-source data related to the frequency response of real buildings during earthquakes. Data collection using citizen science holds major potential to exponentially increase the number of instrumented buildings.

**Figure 1:**
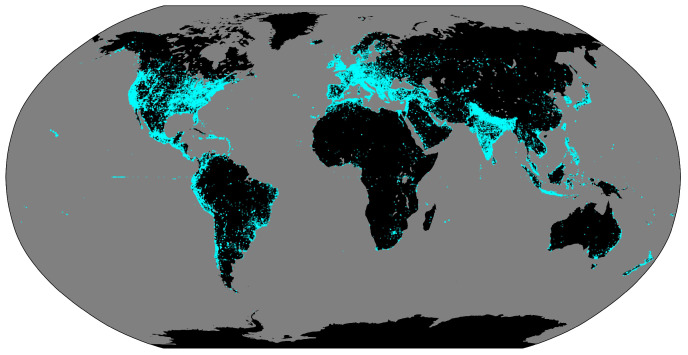
A global map of the distribution of MyShake downloads, marked in cyan points, since the app’s initial release in February of 2016, current as of June 2023.

MyShake is a free app that has been available publicly and globally on Android phones since 2016 and on iPhones since 2019. As of August 2023, MyShake has been downloaded over 2.7 million times by mobile users (Figure 1). While other SHM and earthquake smartphone apps have been launched or are under development (e.g., [7,12,14,15,19]), MyShake is distinct in two ways. First, it employs an auto-collection strategy (described below) that captures the vibrational data without requiring user intervention. Second, MyShake offers additional user-centered features beyond its citizen science functions to encourage broad usership beyond users/researchers specifically motivated to participate in seismology or SHM efforts. Users can access real-time earthquake information, submit and view community damage reports, and obtain safety and preparedness information [20,21]. The app has gained significant traction in the US states of California, Oregon, and Washington, where it delivers ShakeAlert earthquake early warnings [22]. These features not only facilitate the app’s continued growth but also portend a scalable global SHM system that can be implemented at the cost of only app development and server maintenance.

Initial observations of MyShake’s potential for SHM are detailed in Kong et al., 2018 [16], in which phones were set to record vibration continuously at a fixed location on the top floor of a building equipped with a rooftop mechanical shaker. In the present paper, we further validate MyShake’s capabilities in a series of shake table tests, and demonstrate the app’s operation in field conditions. In field conditions, smartphones belonging to private users are placed arbitrarily within a building and spontaneously triggered to record an accelerometer waveform. The uploaded waveform data are then used to extract the structure’s fundamental frequency. Our results, therefore, represent a proof of concept for MyShake’s capability to be leveraged for large-scale near-real-time SHM.

In Section 2 of this paper, we describe the sensor capability of MyShake smartphones and our methodology for collecting and analyzing the frequency content of MyShake’s seismic data. In Section 3, we present the results of a validation experiment performed at a shake table facility. In Section 4, we present real data collected on user smartphones. A discussion of our results and a summary of our conclusions occur in Section 3.3, Section 4.3 and Section 5.

## 2. MyShake Smartphones as Sensors

### 2.1. Sampling Rate

**Figure 2:**
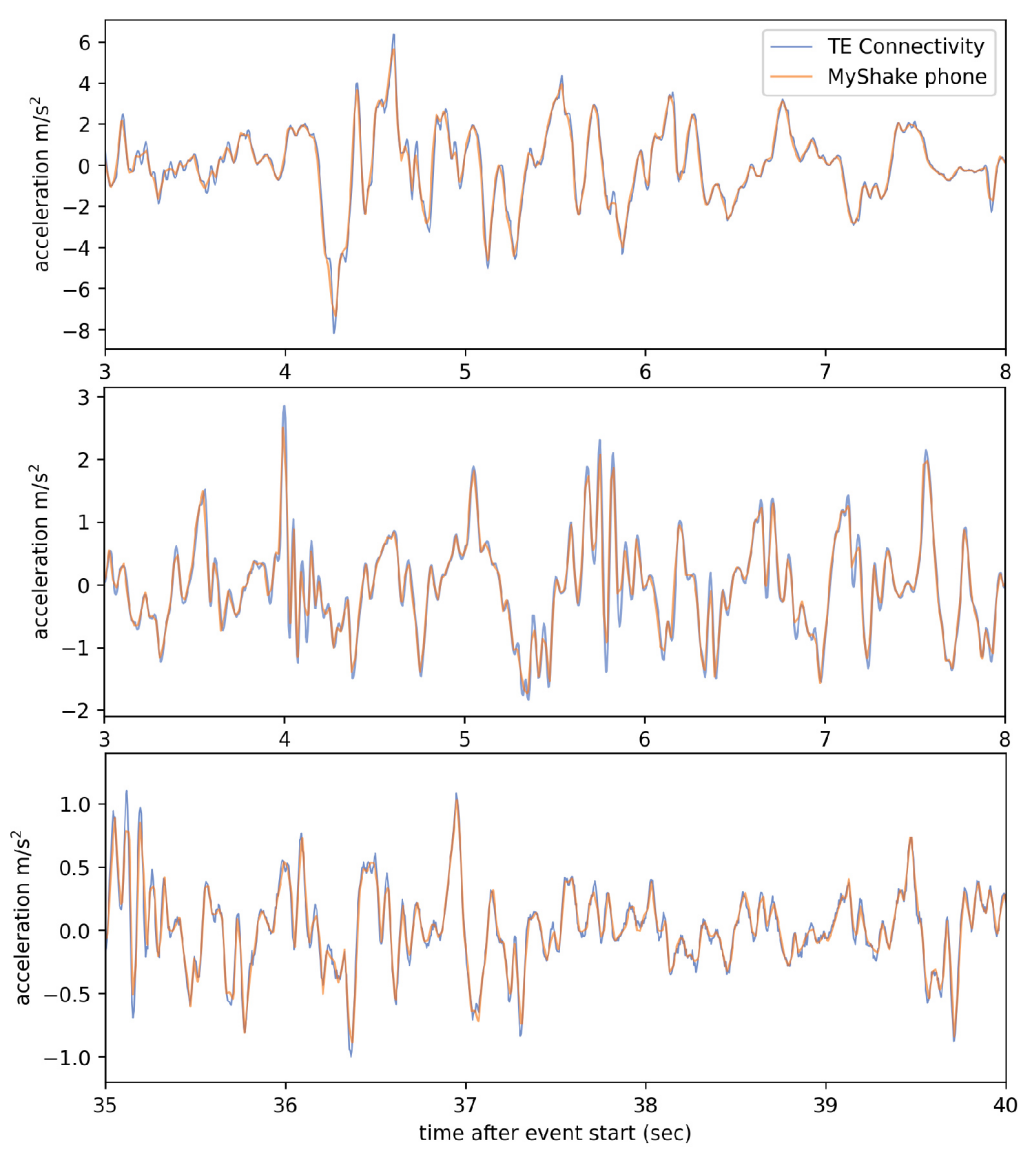
Horizontal component waveforms snippets recorded by phones at 50 Hz compared to those recorded by a co-located high-quality reference sensor (a TE Connectivity brand MEMS accelerometer) at 200 Hz during simulated earthquake motions on a shake table. Phone data have been demeaned and resampled.

MyShake leverages the triaxial MEMS accelerometer built into smartphones to record vibrations. Capabilities and limitations inherent to this hardware relative to a traditional higher quality seismic sensor have been explored previously [23,24,25]. Specifications vary by manufacturer and phone model but, in general, have improved in quality over time. For example, the average sampling rate in accelerometer recordings MyShake made between 2016 and 2018 was 25 Hz. More recent recordings average 50 samples per second. As a result, the Nyquist frequency used to limit phone waveform analysis in this paper will vary based on sample rate (12.5 for the 25 Hz records and 25 for the 50 Hz records). Since we expect the majority of buildings to have a fundamental frequency of about 10 Hz or less, smartphones are quite capable of capturing the information required for characterizing a building’s dynamic characteristics [26]. We can also compare the signal captured at this lower rate to that recorded by a better-quality reference accelerometer—in this case, a TE Connectivity Model 3022 accelerometer—which has a sampling rate of 200 Hz [27]. The lower sampling rate of phones does cause small differences in the observed peak amplitude values, but overall, we find very good agreement between the waveforms (Figure 2).

### 2.2. Noise Floor

**Figure 3:**
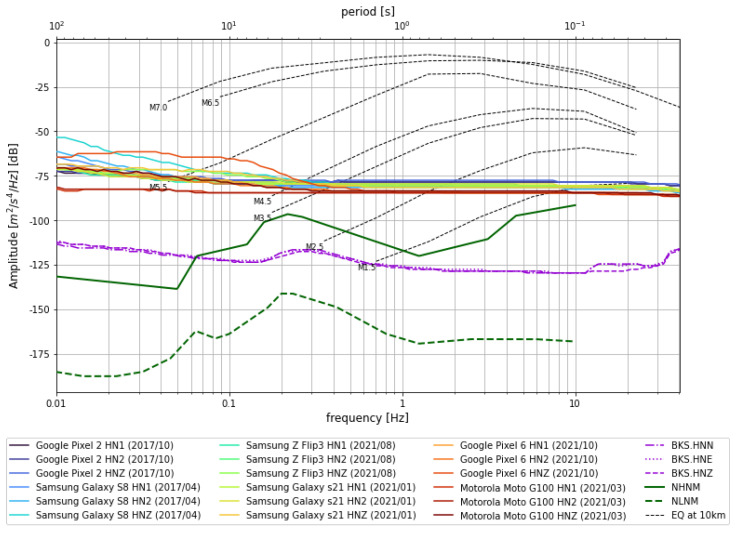
A power spectral density plot that averages over 1 h recordings on three components for several smartphones and the BKS seismic station. The seismic station noise for all components is represented by the dashed and dotted purple lines. The green lines represent the high and low noise model [28].

Smartphone accelerometers typically have a higher noise floor than dedicated seismometers, which reduces sensitivity [7,23]. This was first quantified for MyShake in 2016 with typical smartphones released between 2010 and 2013 [23]. The average internal machine noise amplitude for those phones was around −40 decibels (dB). Here, we again measure the noise floor with more recent phone models. We use 2 smartphones from 2017 (Google Pixel 2 and Samsung Galaxy S8) and 4 from 2021 (Samsung Z Flip, Samsung Galaxy s21, Google Pixel 6, and Motorola Moto G100). All 6 were set on an isolated concrete pier adjacent to the traditional observatory quality seismic station BRK on UC Berkeley’s campus, which is part of the Berkeley Digital Seismic Network (BDSN), and left to record in quiet conditions for 1 h (Figure 3). In this test, we find a ∼35 dB reduction in noise relative to the previous study, with an average amplitude around −75 dB. Smartphone noise from different models has greater variability in low frequencies (about −50 to −80 dB) and less (around −78 to −85 dB) in the high-frequency range.

This noise floor improvement is especially dramatic when put into the context of the dB scale, which expresses power spectral density (PSD) per Hz, relative to 1 (m/s/s)${}^{2}$ (e.g., a power unit). We assume the noise signals are random and uncorrelated. Decibels (for power) is equal to $10log_{10}\left(\frac{power}{power_{ref}}\right)$, and therefore the observed 35 dB reduction in the new experiment equates to a ∼3200 times reduction in noise power. Noise reduction can also be attained via signal stacking, with the amplitude change inversely proportional to $\sqrt{N}$, where *N* is the number of time series. Put another way, the signal from 56 older 2010–2013 phones would need to be stacked in order to reduce the noise by 3200 times and achieve the same noise level as a new model phone. As smartphone technology continues to evolve, we anticipate further improvements to the accelerometer noise floor.

### 2.3. Timestamp

The waveforms collected by phones also depend on the internal clock of the phone to label their timestamps. The MyShake app prompts phones approximately every hour to compare their clocks to a network time protocol (NTP) server and report both the round-trip time required to ping the NTP server and the offset between phone and server clock times. MyShake is unable to change a phone’s clock. Instead, we can interpolate between NTP reports to correct waveform timestamps before analysis. An initial analysis of 6 million NTP reports in 2018 found that phone clocks varied from NTP server times by up to 1.4 s 75% of the time [25]. The difference in reported clock error from one NTP report to the next was within 0.132 s for 75% of samples. We consider this ‘offset change’ an upper boundary on how accurate MyShake timestamps become when NTP reports are used to correct them.

**Figure 4:**
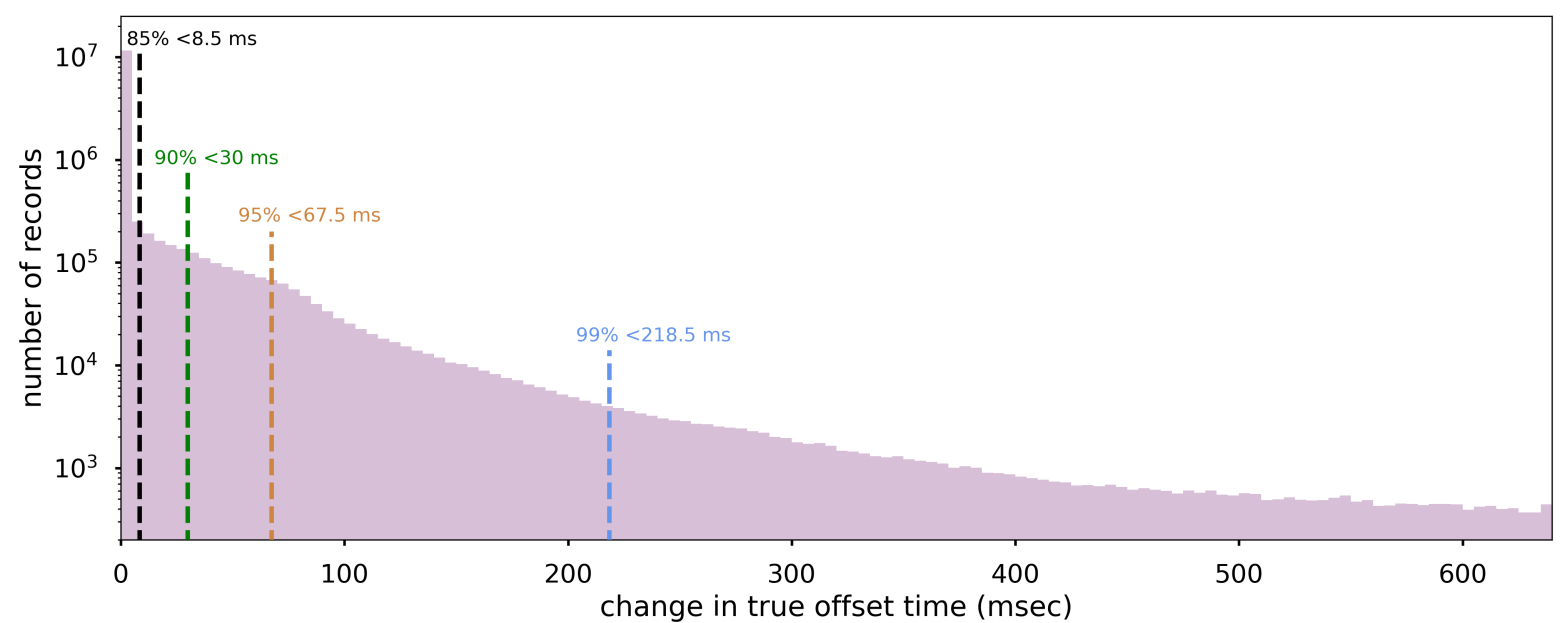
Accuracy of MyShake trigger and waveform timestamps, as represented by the change in reported offsets from NTP server time. Data are extracted from 26 random days of NTP checks between February 2022 and February 2023, resulting in 14 million data points from 58,000 phones. The colored lines mark the 85th, 90th, 95th, and 99th percentiles. Shown is 99.8% of the range. Note that the vertical axis is logarithmic and begins at 10${}^{2}$.

We later updated our 2018 analysis using 14 million new NTP checks recorded on a random selection of 26 days between February 2022 and February 2023. We find that 50% of queries report an internal clock error better than 0.710 s, and 75% were better than 1.23 s. While these absolute offsets are fairly consistent with the 2018 values, the distribution of the errors is improved (Figure 4). In the new analysis, 50% of offsets changed by less than 32 ms (very similar to the 27 ms median in 2018), but the tail is much shorter; 85% of offsets change less than 111 ms, compared to 330 ms in 2018. The typical MyShake waveform is 5 min long. With the target interval between NTP checks set at 1 h, a median deviation of 32 ms spread over the course of 60 min is expected to exert negligible influence on the sampling accuracy within a given waveform. Therefore, we expect no error to be introduced by sampling accuracy to the frequency spectra and the corresponding natural frequency identifications that we discuss in the following sections.

### 2.4. Location

**Figure 5:**
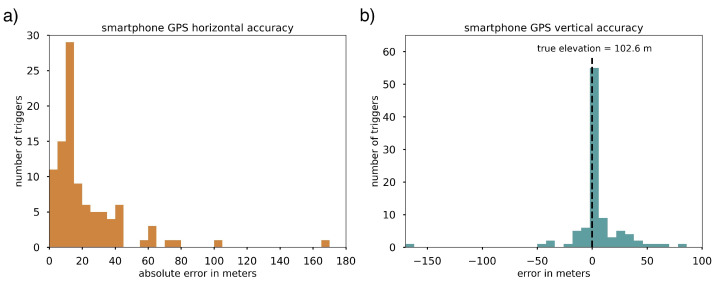
Accuracy of smartphone location using global positioning system (GPS) points reported with MyShake triggers and seismic waveforms, both (**a**) horizontal and (**b**) vertical. Ten phones were placed on a second-floor windowsill facing into a partially sheltered courtyard and periodically prompted to collect a spontaneous trigger and record a waveform. The resulting 98 GPS points cluster closely around the true location, both horizontally and vertically.

An experiment reported in the study of Kong et al., 2019 [25] referenced earlier found 75% of phone GPS location accuracy varied within 28.8 m horizontally and 11.4 m vertically (3–4 stories in a typical building), based on a comparison of phone-reported locations and a ground-truth location (Figure 5; [25]). Phones also self-report estimated accuracy (defined by Android as the radius within which there is a 68% chance the phone is located [29]) with their location data. This tends to be similar but not identical to our measured error; in the same test, the 75th percentile of phone-reported horizontal accuracy was 20.8 m. For the purpose of this study, we restrict our analysis to phones with a self-reported error of 30 m or less, and use waveforms where a circle defined by the provided latitude and longitude and error radius substantially encapsulates the footprint of a building, or if the circle only meaningfully intersects with a single building (e.g., that building is surrounded by enough empty space that phone is not likely to be located anywhere else).

### 2.5. Recording Conditions

The MyShake app records vibrations only when the accelerometer is not already in motion. When a phone is left stationary for 30 min, the app begins monitoring the accelerometer while retaining a one-minute buffer window of data. Phones can both be remotely and internally triggered to record a 5 min waveform (4 min after the trigger in addition to the 1 min buffer) and upload these data to MyShake’s central server for analysis. Internally, the app runs an onboard machine learning algorithm that can detect sudden earthquake-like motion and trigger recording [23]. The MyShake backend server can also trigger ‘ready’ phones to record. All ShakeAlert earthquake early warning alerts sent to MyShake phones trigger recordings, in anticipation of the arrival of shaking. Recordings can also be initiated manually, in anticipation of an event of interest. We utilize this manual triggering function to collect the data described in the shake table test section below. Importantly, MyShake is not currently utilized to make extended (>5 min) continuous recordings out of concern for battery drainage and memory usage on user smartphones.

### 2.6. Fundamental Frequency

Some engineering parameters require the use of time series from sensors in multiple locations within the same structure. One example is the interstory drift ratio, which describes the relative displacement of two consecutive floors over the height between them [19]. For such parameters, we must have high confidence in the accuracy of the timestamps and the vertical location of phones. Because of the uncertainty in the accuracy of absolute timestamps using phone clocks, especially for older records, and the absence of a real-life example of multiple waveforms recorded in the same building during the same event, we focus in this paper on a keystone SHM parameter that can be computed using a single sensor and does not depend on multiphone clock synchronicity: fundamental frequency. In the future, with the development and validation of a robust clock correction method, increased location accuracy, and with a growing stock of MyShake waveforms, it will be possible to explore new parameters.

Fundamental frequency (also called natural or modal frequency) is an essential dynamic characteristic of a building that can be used to qualitatively and quantitatively characterize the response of a building to an earthquake ground motion or other dynamic loading, to update numerical structural analysis models, and to facilitate determination of the presence of damage due to material degradation, environmental conditions or extreme events, such as earthquakes. Damage to a structure (i.e., due to heavy earthquake shaking) generally results in a permanent stiffness reduction of the building and corresponding reduction to the modal frequency [2,30].

For identifying the fundamental frequency using MyShake waveforms, we use multitaper spectral analysis, as developed by Prieto, 2022 [31]. This method is tailored for geophysical data to suppress noise by applying orthogonal tapers to overlapping windows of the time series before estimating the power spectral density.

In SHM, fundamental frequency is commonly measured by identifying the amplification in the structure’s response for each frequency in the input forcing signal through the use of a transfer function. Essentially, a division of a spectrum recorded higher up in a structure by the spectrum of the motion input to the base, transfer functions require at least two measurements to be made per structure. Because our ultimate goal is to measure structural behavior using smartphones whose locations we cannot control, we focus on methods that require only a single waveform measurement in any location in the building, i.e., output-only methods [32].

In traditional SHM, baseline measurements of structural health can be made using ambient noise. However, due to the intrinsic noise levels associated with smartphone accelerometers, we rely on small input motions to excite the modal frequency to a level we can measure. In this study, we focus on earthquake motions as the input excitation. First is a controlled validation test, in which phones were co-located with higher quality MEMS sensors on a steel structure built atop a mechanical shake table. Second are ‘real-world’ examples using small earthquake motions recorded by user phones. Since some transient effects can occur during a strong excitation, we particularly prioritize measuring free vibrations that persist after the input motions cease.

## 3. Shake Table Test

### 3.1. Methods

**Figure 6:**
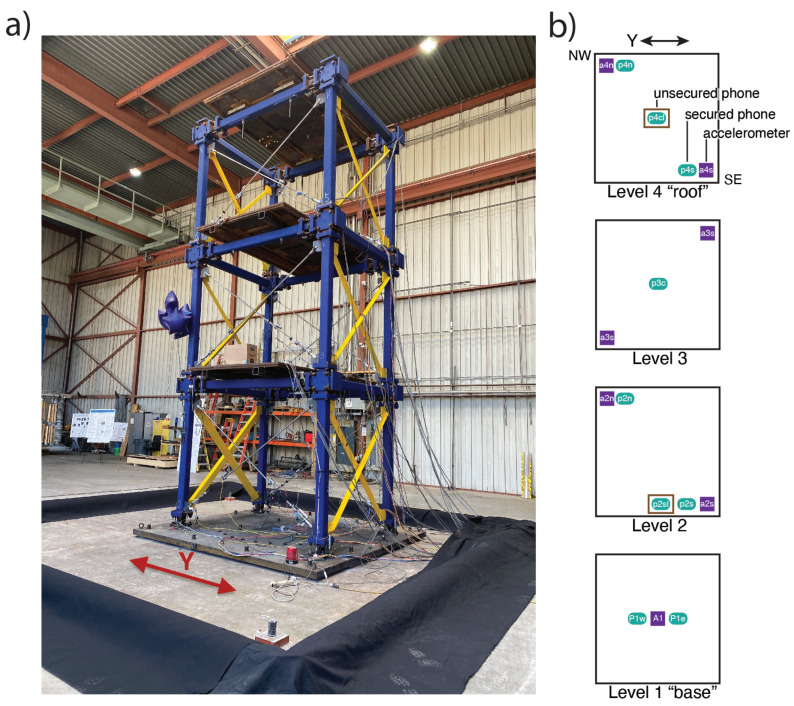
A 3-story test structure built upon the PEER shake table. (**a**) A photograph of the structure and accompanying measurement equipment. The thin silver cross-braces that can be seen on the face of the structure closest to the viewer are the SMA braces; motion was applied in their direction, as indicated by the red arrow. (**b**) A layout, not to scale, of the structure to demonstrate where the sensors were located. Violet squares represent mounted TE Connectivity accelerometers. Turquoise ovals represent phones. ‘Secured’ means the phone was affixed directly to the structure, and ‘unsecured’ means the phone was placed on the structure with no adhesive. To keep unsecured phones from flying off the structure, a cardboard box like the one seen in the photograph on Level 2 was secured over it.

**Figure 7:**
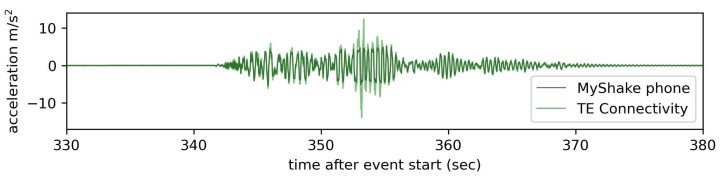
The waveform during which the unsecured phone at the test structure’s top level appears to have slid during high-input accelerations (above a threshold of ∼5 m/s${}^{2}$), creating a clipping effect in the record. The black line represents the unsecured phone, and the green is the same motion captured by fixed traditional accelerometers. This waveform is the third in a set of three consecutive applications of the 1999 Kocaeli earthquake in Türkiye; the timestamps are relative to the start of the first motion.

A three-story steel frame with replaceable SMA (shape memory alloy) braces providing self-centering and energy dissipation capabilities was tested on the Pacific Earthquake Engineering Research Center (PEER) 6-DOF (degrees of freedom) shake table (Figure 6a). To record the structural response, each floor of the structure was instrumented with TE Connectivity 3022 MEMS accelerometers [27]. These sensors have a dynamic range of ±10 g and a sensitivity of 3–6 mV/g. We placed nine phones running the MyShake app alongside, in the arrangement depicted in Figure 6b. Most phones were firmly attached to the structure, in order to compare them directly with the conventional sensors. Two were placed on the structure but not adhered, to simulate more ‘natural’ circumstances. The unsecured phones were put in basic soft plastic cases, and a box was taped over each such that they could slide within the box confines but not be flung off the structure entirely. Previous shake table tests have demonstrated that phone sliding is recognizable in waveforms by its resemblance to clipping [23], and can be visually identified during processing. In the highest-amplitude motions applied in this test, the unsecured phone on the structure’s top level appears to clip when subjected to greater than ∼0.5 g (5 m/s${}^{2}$) motion (Figure 7). This threshold for sliding is somewhat higher than was observed in the previous Kong et al., 2016 [23] study, potentially due to the friction provided by the soft plastic cases. Notably, no observations of sliding appear to noticeably affect the captured frequency of the motion; the phone slides during high acceleration, clipping the waveform, and begins capturing motion faithfully again as soon as acceleration drops again.

The shake table that was used in the tests can produce motions in three translations and three rotational degrees of freedom; however, because the main interest was to explore the performance of the SMA braces, ground motion inputs were only applied in the direction parallel to these braces (indicated by the double-headed arrows in Figure 6). As such, the time series and frequency plots we show in this section are all based on this single component of horizontal motion. It is noted that stiff braces (painted yellow) were placed in the orthogonal direction to completely separate the natural frequencies in the two orthogonal directions.

A series of motions was applied to the test structure, including ground motions from the 1999 Kocaeli/Izmit earthquake in Türkiye and the 2006 Kobe earthquake in Japan. In some cases, the same motions were applied multiple times at increasing amplitude levels.

**Figure 8:**
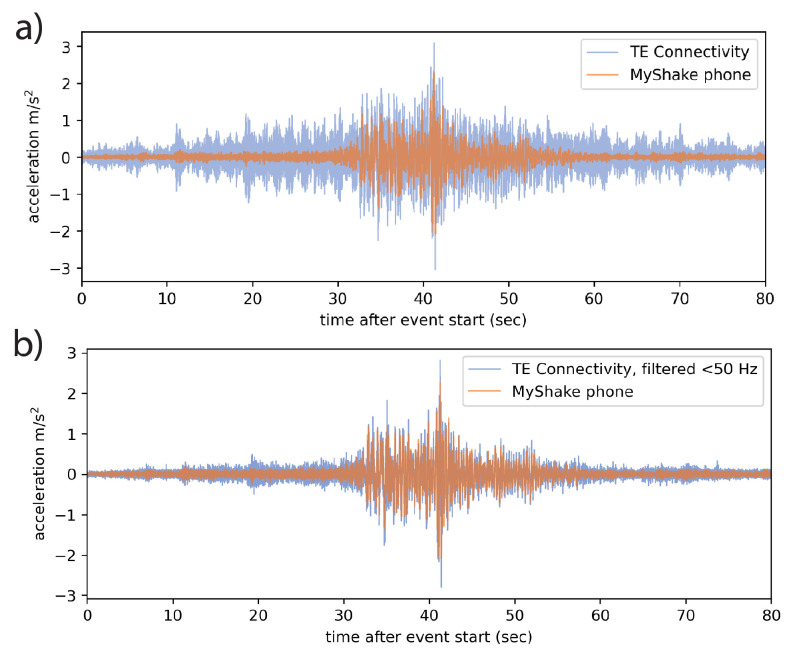
MyShake acceleration recordings in orange overprinting TE Connectivity acceleration recordings in blue, both from the roof of the test structure. (**a**) Unfiltered 200 Hz TE data displays a high amplitude, high-frequency signal during the earthquake motions. (**b**) Once low-pass filtered below 50 Hz, the TE and MyShake data resemble each other more closely.

The conventional sensors had a 200 Hz sampling rate and the sensors located at the second level and above recorded high-frequency vibrations during earthquake motions that were not captured by phones with their average 50 Hz sample rate (Figure 8a). To provide a more direct comparison between the behavior and record quality of the two sensor types, the TE records were filtered before analysis with a 50 Hz low-pass filter (Figure 8b). This filtering isolates the frequency band of interest (<25 Hz) as there are no natural frequencies of the tested structure above 25 Hz that provide contributions to the dynamic response of the structure. After this filtering, there is a good match between the TE and the MyShake records.

Using multitaper spectrum analysis [31,33], we transform three Kocaeli runs, applied consecutively and recorded by phones and the conventional sensors, into the power spectral density (PSD) space. We use 20 s windows moving in 1 s increments, which provided good resolution in multiple test cases. Based on the parameters used in our analysis, we can reliably resolve modal frequencies with separation greater than about 0.4 Hz. To simulate an equivalent to the motion experienced by the phone at the center of the structure, we average the signals recorded by two conventional sensors installed on opposing corners of the same level.

### 3.2. Results

**Figure 9:**
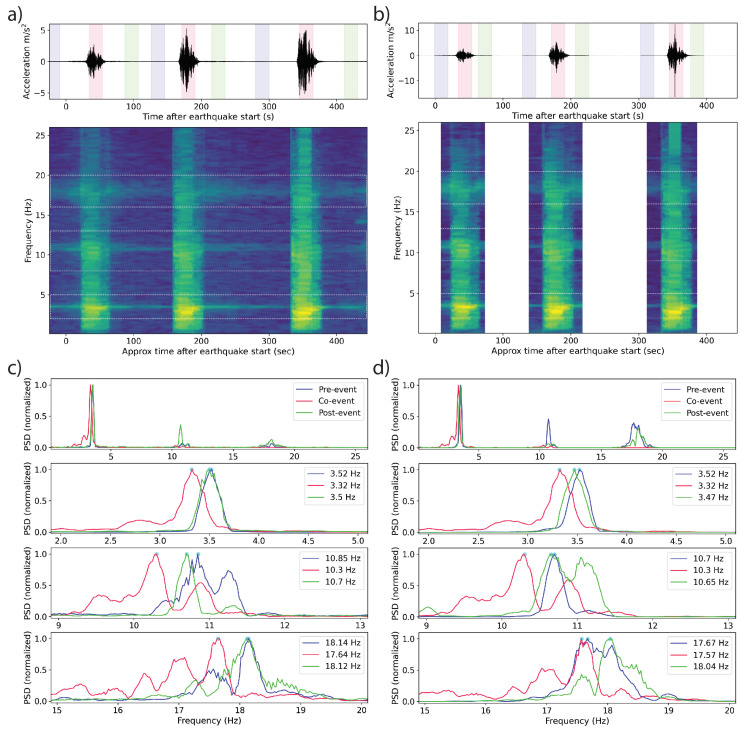
Measurements made over three runs of the 1999 Kocaeli earthquake on a shake table as recorded by both a MyShake phone and a reference TE Connectivity Model 3022 MEMS sensor on the top of a 3-story steel structure. (**a**) The MyShake waveform and corresponding frequency spectrum. Three modes are clear throughout the time series, brightening and dipping somewhat during the earthquake motions. The first three colored bands correspond to the windows in the time series represented in (**c**). (**b**) The MEMS sensor filtered and averaged waveform and corresponding frequency spectrum. The same modes present in the MyShake data are visible. (**c**) Pseudospectral density (PSD) plots from windows of the MyShake accelerations recorded during the first earthquake run visible in the waveform. Blue lines represent the PSD of the structure excited by table vibration before the earthquake. Red lines represent the strongest PSD observed during earthquake shaking, and green lines represent the PSD after the earthquake is over. (**d**) Corresponding PSD plots for the reference measurements, using the first and final windows for the pre- and post-seismic measurements, respectively.

Sample frequency spectrum and associated PSD plots from the top of the structure, where the dynamic response is greatest, are shown in Figure 9. For each of the three Kocaeli motions, frequencies of the first three modes are identified before, during, and after the motion (denoted as pre-event, co-event, and post-event, respectively) using PSD plots of the MyShake and conventional accelerometer recordings. The segments were selected by visual inspection. Note that there are still small vibrations of the shake table in the times between the application of the ground motions due to the control dynamics of the table when the actuators are still pressurized to keep the table at a constant position. The first and second modes appear strongly for both the phone and TE sensors, and are consistent between them, especially pre- and post-seismic motions. Because the conventional data were not recorded continuously, we selected the first and final windows to represent the pre- and post-seismic PSDs. Across the three tests, the pre-earthquake baseline fundamental frequency varied between 3.50 and 3.52 Hz (0.284–0.286 s period). The secondary baseline frequency varies between 10.7 and 10.87 Hz (0.092–0.093 s period). The results were also consistent with the baseline frequencies observed in the later Kobe tests, varying between 3.42 and 3.50 Hz (0.286–0.292 s period) for the first mode and 9.00 and 10.97 Hz (0.091–0.111 s period) for the second. A summary of results can be viewed in Table 1. The periods identified using the TE and MyShake accelerometers are in general quite close to each other.

### 3.3. Discussion

The SMA braces utilized in this test provided restoring force capability only under tensile forces and did not provide any stiffness or strength under compression. Although some pre-stress is provided to maintain the tension forces, the braces were disengaged at instances during the shaking in most of the tests, reducing the stiffness of the structure, resulting in a modal frequency reduction by as much as 22%. In a typical structure without similarly engineered components, the same signal would indicate that the structure has lost stiffness by other means, e.g., damage [2,30]. When the earthquake motion ceased during the test, the braces disengaged, returning the structure to its original stiffness. In some cases, the bolts securing the rods loosened slightly, such that the stiffness did not fully recover. An example can be seen in Figure 9d, where the pre-event modal frequency is slightly higher than the post-event value. The failure of a structure to recover to its original stiffness indicates inelastic deformation occurred. Because the rods employed in the shake table test were experimental and their tensioning varied between tests, we cannot interpret the frequency changes we observed beyond simply ‘indicated damage’. However, the clear and repeated co-seismic deviation and post-seismic recovery (both partial and complete), visible in phone and traditional records alike, marks a first-order validation that phones are capable of capturing subtle and important changes in modal frequencies and therefore in structural health information.

## 4. Citizen Science Measurements

Having demonstrated that we can use waveform data recorded by the MyShake app to measure the modal frequencies (and changes to them) of a building, in the context of a shake table experiment, we now look to measure the modal frequencies using MyShake data from real buildings as provided by the crowd-sourced MyShake data. This brings additional challenges. First, we have only the reported GPS location and barometric altitude to locate the phones. As previously discussed, this is accurate enough to locate the phone inside a building, but not accurate enough to locate the phone in a specific location within that building. The exception is when the structure is very tall, in which case the altitude could be used to make generalized statements like ‘bottom’, ‘middle’, or ‘top’. Similarly, we do not know the orientation of the phone relative to the axes of the structure.

### 4.1. Methods

**Figure 10:**
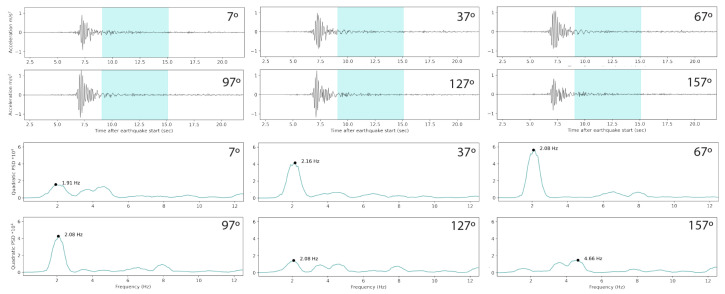
An example of waveform rotations in 30° increments in the horizontal plane, demonstrating the effects of changing orientations in both the time and frequency domain. The phone recording was located in an 8-story hotel in Northern California, and located 16 km from a Mw 4.4 earthquake. The spectra are computed from the free vibration portion of the waveform, as highlighted in cyan. The maximum spectral peak corresponding to a frequency of 2.08 Hz is found at 67° from the phone’s *y*-axis, and a smaller secondary frequency of ∼4.7 Hz is found in the perpendicular direction, potentially corresponding to the building’s second axis.

For a first-order validation experiment, we begin with multistory buildings with a simple rectangular footprint. We expect the first mode to be most strongly expressed in the direction of one of the building’s axes and seek to rotate the waveforms into alignment. First, all three orthogonal components are rotated to bring the mean of two components (which we name X and Y) as close as possible to 0, such that the Z component is in the direction of gravity. Second, the newly identified horizontals are incrementally rotated, and the coda (the free vibration part of the motion) is transformed to find the orientation in which the lowest frequency peak is at its strongest, which implies it is aligned with one of the horizontal axes of the building. It is noted that it is not possible to identify for certain which horizontal axis it is, although general rules of thumb and engineering judgment can be used to make an educated guess. An example of how rotations affect the resultant frequency spectra is in Figure 10.

While earthquake shaking results in the motion of a building predominantly at its natural frequency, the frequency spectrum of a co-seismic waveform will also include the frequency content of the source. Lacking an input ground motion record that could be used to calculate a transfer function, we instead must measure the fundamental frequency of the building using the free vibration, which occurs after the strong motion is over and which is dominated by the building’s dynamic characteristics. This transition is easily identifiable when the waveform and the power spectrum are viewed in parallel.

### 4.2. Results

**Figure 11:**
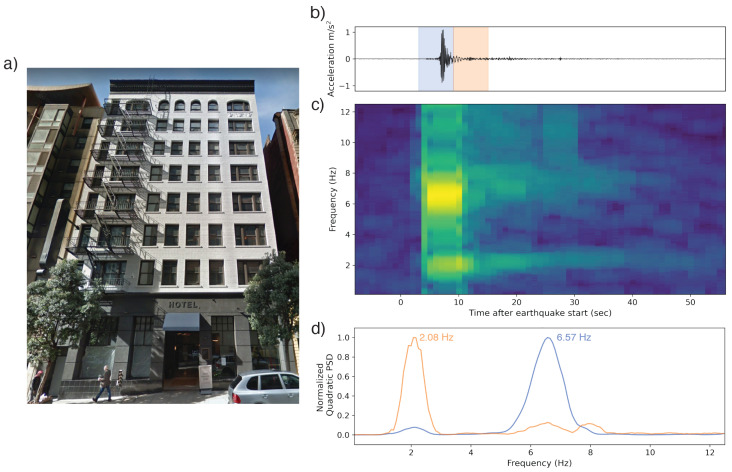
(**a**) An 8-story hotel in the California Bay Area whose fundamental frequency was captured by MyShake during the Mw 4.4 Berkeley earthquake originating 16 km away. (**b**) The waveform collected by the phone. The blue and orange overprints represent the windows over which the power spectral density (PSD) plots in (**d**) were computed. (**c**) The corresponding time–frequency spectrum computed using multitaper analysis. Spectra are centered relative to their corresponding window in the accelerogram. The fundamental frequency of 2.08 Hz is visible in the spectra for the entire duration, including in the free vibration, though it is dwarfed in strength during the body of the earthquake by a peak at 6.57 Hz, approximately 3× the fundamental and potentially representing the building’s second mode. (**d**) Normalized PSDs for two of the 6 s windows identifying the dominant frequencies in each.

**Figure 12:**
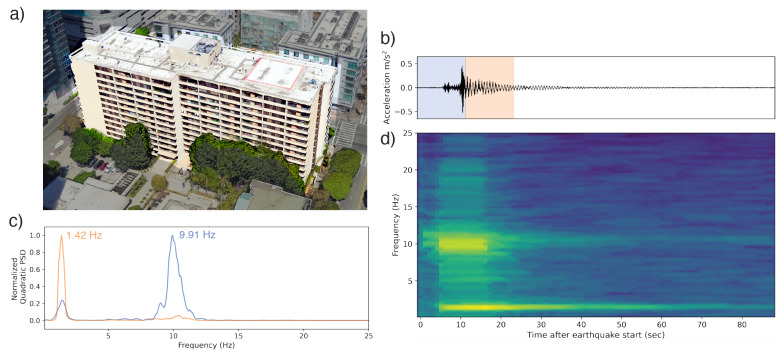
(**a**) A 14-story condominium complex in southern California, which recorded a waveform of a Mw 4.3 earthquake 24 km away. The 3D rendering is sourced from GoogleEarth with a darkening mask applied to highlight the building of interest. (**b**) The waveform collected by the phone. The blue and orange overprints represent the 12 s windows whose PSDs are represented in (**d**). (**c**) Normalized PSDs for two of the 12 s windows identifying the dominant frequencies in each. (**d**) The time–frequency spectrum computed using multitaper analysis. Spectra are centered relative to their corresponding window in the accelerogram. A peak at 9.91 Hz is visible during the initial body of earthquake shaking, fading in strength later relative to a natural frequency peak at 1.42 Hz.

**Figure 13:**
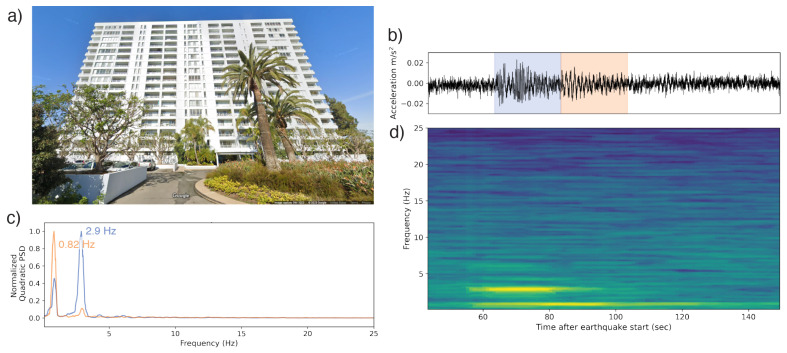
(**a**) An 18-story apartment building in southern California, which recorded a waveform of a Mw 5.4 earthquake 212 km away. (**b**) The waveform collected by the phone. The blue and orange overprints represent the 20 s windows whose PSDs are represented in (**d**). (**c**) Normalized PSDs for two of the 20 s windows identifying the dominant frequencies in each. (**d**) The time–frequency spectrum computed using multitaper analysis. Spectra are centered relative to their corresponding window in the accelerogram. A peak at 2.9 Hz is visible during the initial body of earthquake shaking, fading in strength later relative to a natural frequency peak at 0.82 Hz. Being ∼3× the latter, it is possible the 2.9 Hz peak is a secondary mode.

**Figure 14:**
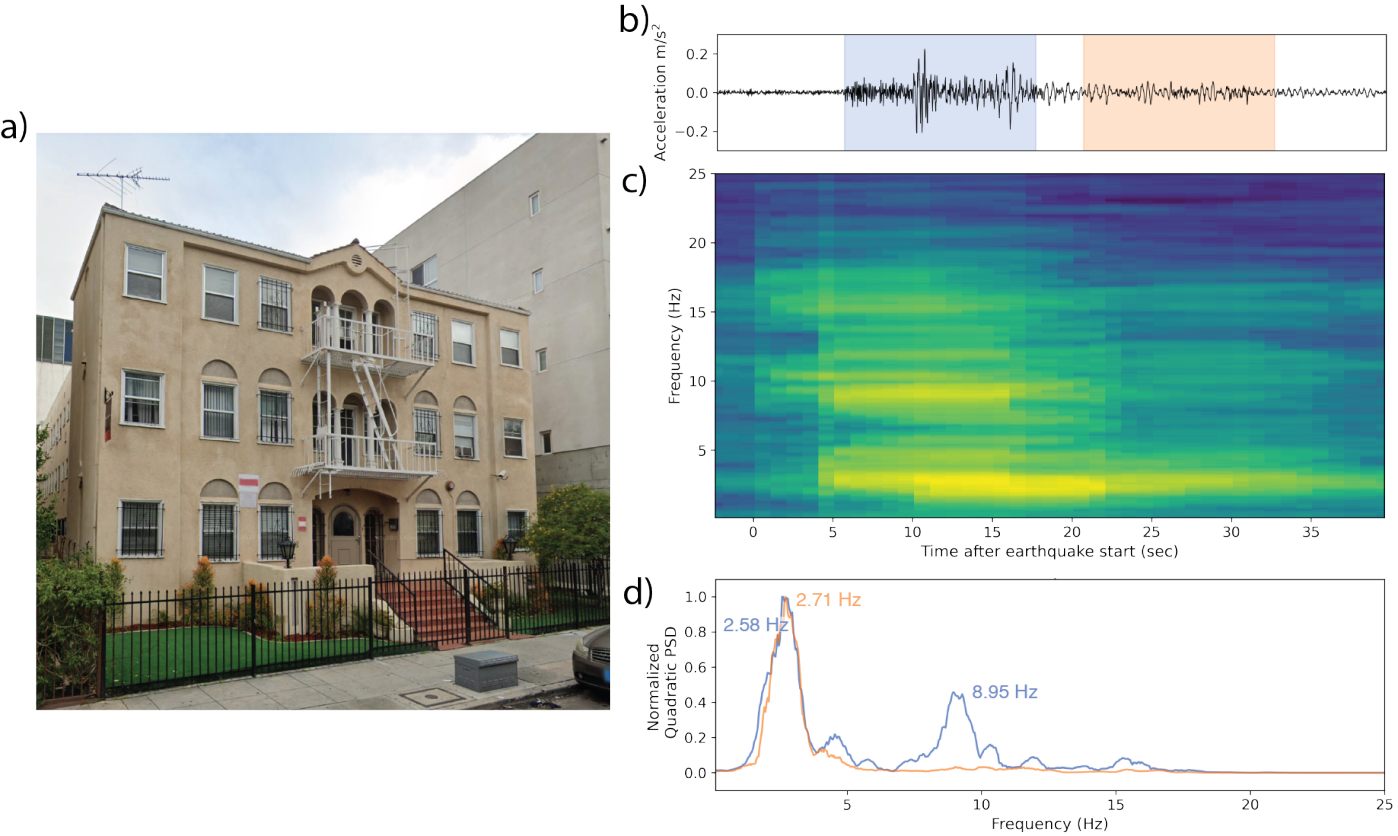
(**a**) A 3-story apartment building in southern California, which recorded a waveform of a Mw 4.3 earthquake 25 km away. (**b**) The waveform collected by the phone. The blue and orange overprints represent the 10 s windows whose PSDs are represented in (**d**). (**c**) The corresponding time–frequency spectrum computed using multitaper analysis. Spectra are centered relative to their corresponding window in the accelerogram. The strongest peak throughout occurs at 2.5–2.7 Hz (0.37–0.4 s). During the body of the earthquake, there is a second peak at 8.95 Hz (0.112 s), which later subsides. (**d**) Normalized PSDs for two of the 10 s windows identifying the dominant frequencies in each.

**Figure 15:**
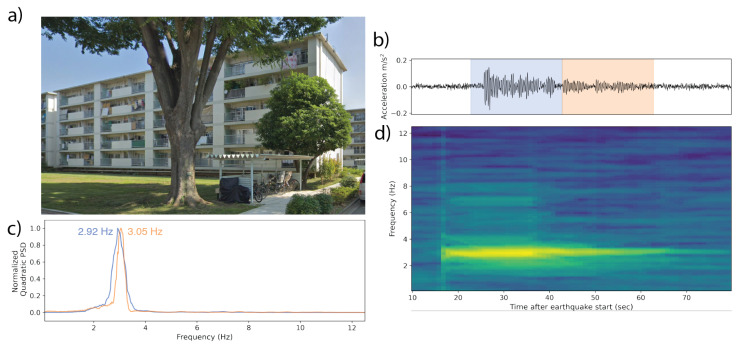
(**a**) A 5-story apartment building in Japan, in which MyShake recorded the Mwr 4.7 Tsukuba earthquake originating 73 km away. (**b**) The waveform collected by the phone. The p-wave arrives at ∼12 s. A dominant frequency becomes visible after the start of the s-wave and persists for at least 50 s. The blue and orange overprints represent the windows over which the power spectral density (PSD) plots in (**d**) were computed. (**c**) Normalized PSDs for two of the 20 s windows identifying the dominant frequencies in each. (**d**) The time–frequency spectrum computed using multitaper analysis. Spectra are centered relative to their corresponding window in the accelerogram. A single frequency peak at ∼3 Hz is visible across the majority of the spectra.

**Figure 16:**
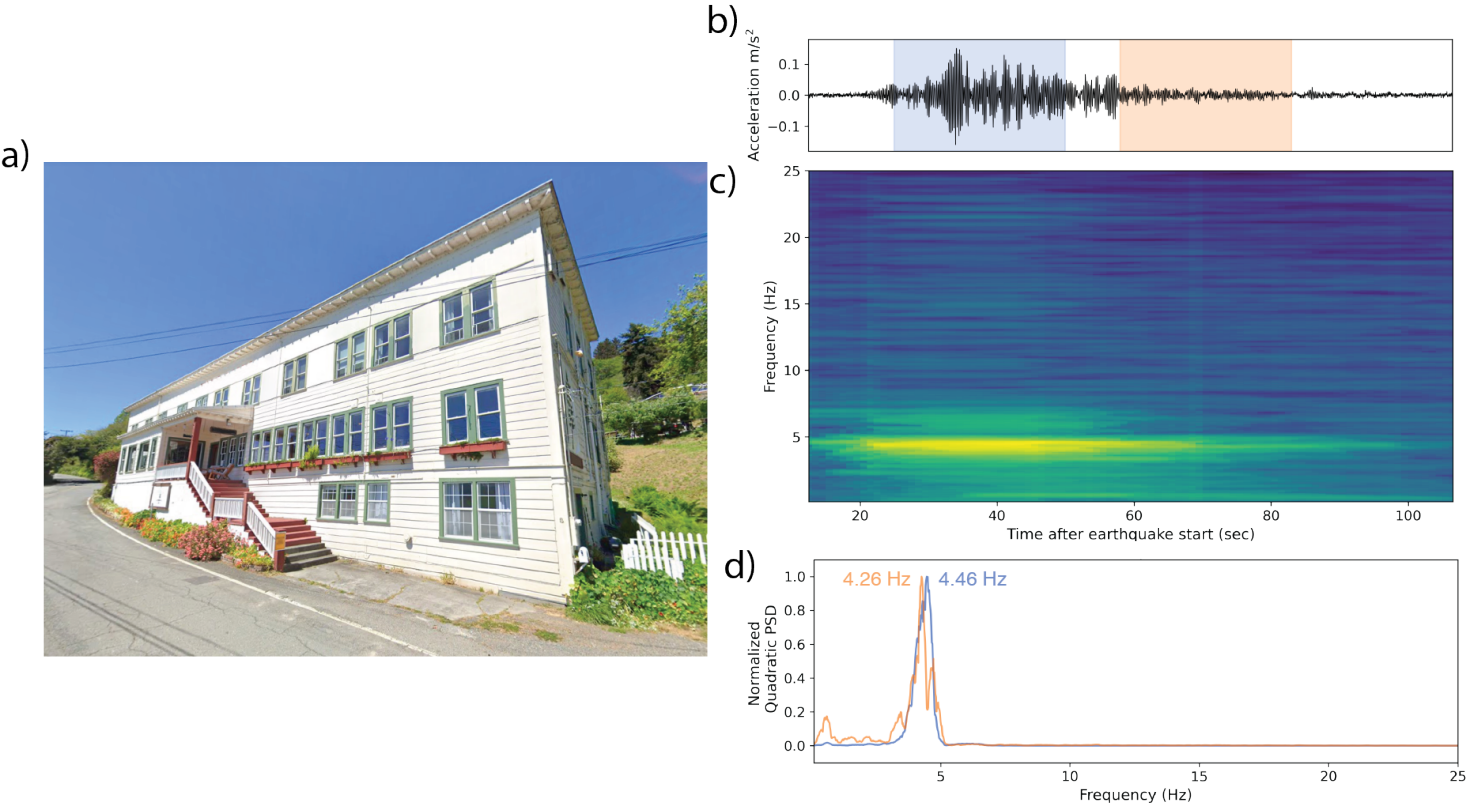
(**a**) A 3-story hotel in Northern California in which a MyShake phone recorded the Mw 6.4 Ferndale earthquake 117 km away. (**b**) The waveform collected by the phone. The blue and orange overprints represent the 25 s windows over which the power spectral density (PSD) plots in (**d**) were computed. (**c**) The corresponding time–frequency spectrum computed using multitaper analysis. Spectra are centered relative to their corresponding window in the accelerogram. A dominant frequency peak at 4.25–4.45 Hz is visible across the majority of the spectra. (**d**) Normalized PSDs for two of the 25 s windows identifying the dominant frequencies in each.

**Figure 17:**
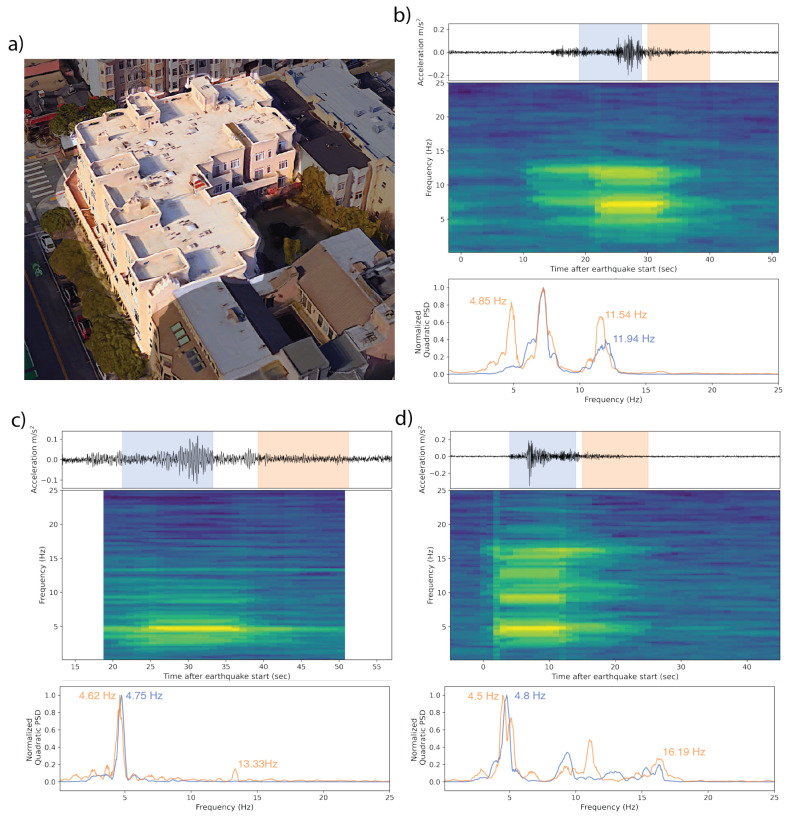
(**a**) A 5-story apartment building in the California Bay Area in which MyShake recorded a waveform in three earthquakes. The 3D aerial rendering is sourced from Google Earth, with a darkening mask applied to highlight the building of interest. (**b**) The waveform and spectra collected in the Ml 4.3 Santa Rosa earthquake 80 km away. The blue and orange waveform overprints represent the 10 s windows over which the normalized PSDs in the bottom panel were computed. The time–frequency spectrum in the center panel shows two dominant frequencies during the body of the earthquake and a third gaining relative prominence as shaking subsides. (**c**) The waveform and spectra collected in the Mw 5.1 Alum Rock earthquake 85 km away. Normalized PSDs are computed using the highlighted 12 s windows. The time–frequency spectrum shows a single dominant and persistent frequency peak excited for this event. (**d**) The waveform and spectra collected in the Mw 3.6 El Cerrito event 17 km away. Both the highlighted windows and the time–frequency plot as a whole show a main, slightly time-variant frequency at ∼4.65, and a few more mobile higher frequency peaks.

We present six real-world examples of MyShake recordings from user phones in earthquakes from California and Japan (Table 2, Figure 11, Figure 12, Figure 13, Figure 14, Figure 15 and Figure 16). Each is a rectangular structure varying between 3 and 18 stories tall. Images of each building are sourced from Google Street View and Google Earth 3D buildings. Additionally, we present the example of an irregularly shaped five-story building, whose response was captured in three different earthquakes (Figure 17). While a single-response spectrum from this building can be interpreted with some ambiguity, repeat measurements across earthquake sources of varying magnitudes (M 3.6, 4.3, 5.1) and varying path effects (a 150 deg spread in backazimuths) can be used to confirm that the approach of using the free vibration response is effective in capturing the building periods.

For each structure, a sample window of the body of the earthquake and a window dominated by free vibration are highlighted, demonstrating the time-based spectral variation and identifying the frequency peaks of each. Window size and number of tapers (and the corresponding selection of a time-bandwidth product) used in the multitaper analysis scale are based on earthquake duration [33]. For each record, we identify the lowest frequency of the strong peaks as the likely natural resonance of the structure. These values are evaluated in the Discussion section (Section 4.3).

#### 4.2.1. Taller Structures

Observations made in an 8-story hotel during the Mw 4.4 Berkeley earthquake and in a 14-story condominium complex during the Mw 4.3 Carson earthquake both provide good examples of buildings first experiencing forced vibrations from an earthquake, followed by a period of free vibration where the motion is dominated by the free oscillation of the building (Figure 11 and Figure 12). This transition is visible in both the waveforms and the time–frequency plots. For the eight-story hotel, a frequency of 6.57 Hz (0.152 s period) dominates during the main earthquake shaking. When the longer period oscillations start, the dominant frequency is 2.08 Hz (0.481 s period), which is approximately consistent with the natural frequency expected for a structure this size. Being approximately three times the fundamental frequency, it is possible that the 6.57 Hz peak represents the building’s secondary mode. We hypothesize that the higher frequency mode during the earthquake could be a result of the ground motion exciting the building’s second mode, but the free vibration more strongly excited the fundamental mode, as expected for a medium-rise building.

For the 14-story condominium complex, the strongest frequency excited by the earthquake’s initial motion is 9.91 Hz (0.101 s). Following the main pulse of earthquake energy, a longer period oscillation (1.42 Hz or 0.704 s period) begins. As 9.91 Hz is so much higher than the lowest frequency peak of 1.42 Hz, it is possible it represents the third, not second, mode of the structure.

The tallest structure for which we have a good observation is an 18-story apartment building in southern California, whose modal frequency was excited by a Mw 5.4 earthquake during the Ridgecrest earthquake sequence 212 km away (Figure 13). Although the waveform signal is much diminished by attenuation, good-quality spectra can still be obtained. In it, two dominant frequencies are visible: one at 2.9 Hz (0.345 s in period) and another, persisting longer, at 0.82 Hz (1.22 s period). The lower frequency peak is roughly consistent with what would be expected of a building this size. The higher frequency, being three times the lower, potentially indicates a second mode that was excited during the earthquake excitation.

#### 4.2.2. Shorter Structures

The Mw 4.3 Carson earthquake was responsible for a second observation of a three-story apartment building 25 km from the epicenter (Figure 14). This structure demonstrates two, possibly three strong frequency peaks during the main earthquake shaking, with the higher peaks (8.95 Hz and 15.24 Hz) subsiding and giving way to a single lower dominant frequency (2.71 Hz, or 0.369 s period) in the coda of the waveform.

In the case of observations made in a five-story apartment building in Japan, which was shaken by a Mwr 4.7 earthquake 73 km away, and a three-story hotel in Northern California, which was shaken by a Mw 6.4 earthquake 117 km away, a single dominant frequency is present throughout the time series (Figure 15 and Figure 16). In the apartment building, the frequency is 3 Hz (0.33 s period), and in the hotel, it is 4.2–4.5 Hz (0.24–0.22 s). Occurrence of the same frequency during earthquake shaking and the free vibration is a strong indication that these frequencies are the fundamental natural frequencies of the buildings, and during the earthquake shaking, the building response is directly governed by the fundamental mode.

#### 4.2.3. Repeat Measurement

A MyShake waveform was collected in three earthquakes from the same L-shaped five-story apartment building over the course of four months in 2022 (Figure 17a). Being asymmetric in shape, it is more challenging to gauge the reasonableness of any given modal frequency measurement. Repetition, therefore, can serve as a form of verification. In the first earthquake, an Ml 4.3 event in Santa Rosa 80 km from the building, two frequencies peaked coseismically, and a third arose as shaking subsided. The lowest of these was 4.85 Hz (Figure 17b). In the Mw 5.1 Alum Rock earthquake 85 km away in the opposite azimuthal direction, a single primary frequency between 4.6 and 4.75 Hz was excited throughout the earthquake and coda (Figure 17c). The closer, smaller Mw 3.6 El Cerrito earthquake (17 km away) excited two main frequencies coseismically. The primary peak at 4.8 Hz decreased slightly into the coda, down to 4.5 Hz, while the second frequency became more apparent during the coda at 16.19 Hz (Figure 17d). The variation in primary frequency over the three earthquake codas is, therefore, 4.85–4.5 Hz (0.206–0.222 s period), decreasing slightly with each successive earthquake.

### 4.3. Discussion

We establish in a controlled shake table test that the modal frequencies identified by MyShake and traditional MEMS accelerometers are quite similar before, during, and after the ground motion shaking. With a sufficient signal-to-noise ratio, a clear frequency spectrum can be derived even without a transfer function. With this in mind, we extract modal frequencies from several buildings in which MyShake phones recorded a small or distant earthquake. In some cases, we also observed frequency peaks at approximately 3 times the first. These we interpret as possible observations of secondary frequencies.

It is important to evaluate whether the modal frequencies we extracted from our observations are reasonable for the structures in which they were recorded. For this purpose, we make use of the standards set by the American Society for Civil Engineers (ASCE). In ASCE 7-22 standards, two simple, empirically derived formulas are provided for estimating a structure’s fundamental period when only a little information about the structure is known. ASCE 7-22’s Eq 12.8-8, replicated here as Equation (1), is based on structure height and framing type. The commentary states that this estimate is deliberately conservative, and tends to underestimate the true period.

(1) $$T_{a}=C_{t}h^{x}$$

where $T_{a}$ is the approximate fundamental period in seconds, and *h* is the height of the building in feet. The coefficient $C_{t}$ and exponent *x* are provided in Table 3. We add to the ASCE values an additional option for wood frames from Camelo, 2003 [34], which was derived from a series of shake table experiments and performed fairly well for a wider range of building heights in Hafeez et al., 2019 [35]. Goel and Chopra, 1998 [36] also found ASCE’s variables for ‘other’ to apply well to structures with concrete shear walls. Some structures can also exhibit higher order modes at odd-integer multiples of the first.

ASCE 7-22 Eq 12.8-9, replicated here as Equation (2), supplies a quick estimate for steel or concrete moment frames and is also empirically derived. In this, *n* is the number of floors and cannot be larger than 12. The fundamental frequency $\omega_{n}$ (in Hz) is equal to 1/T.

(2) $$T_{a}=0.10n\;and\;\omega_{n}=10/n\;for\;n\leq 12$$

Using these estimates, we can then compare the frequency peaks derived from our observations to computed estimates of what we might expect from a simple model of each structure in Table 4. Our knowledge of the buildings is limited to what is logged in public databases like OSM buildings (https://osmbuildings.org, accessed on 11 August 2023) and what can be visually determined from Google Earth imagery. Therefore, in many cases, we do not know the framing material with certainty, and instead compare our observations with multiple possible estimates.

All observation-based modal frequencies fall within 41% of at least one estimate. Most observation values are higher than the estimates computed using Equation (1) and the variables in Table 3. This is likely due, at least in part, to the presence of shear walls, which Goel and Chopra, 1997 and 1998 [36,37] found contributed significantly to stiffness for accelerations <0.15 g (1.47 m/s${}^{2}$). As visible in Figure 11, Figure 12, Figure 13, Figure 14, Figure 15, Figure 16 and Figure 17, all accelerations used in the analysis of real structures fall well below this threshold. The eight-story hotel and three-story apartment building records give modal frequency measurements that are somewhat lower than the Camelo, 2003 [34] based values for wood-framed buildings. The closest match between observed and estimated (3% difference in frequency) occurs for the three-story hotel, which is within the anticipated error range of the measurement.

While most of our real-structure analysis focused on simple, rectangular framed buildings, we showed an example of an L-shaped three-story apartment building, for which we obtained three measurements in three different earthquakes over the course of 4 months. These observation-based modal frequencies match best with the wood-based estimate. Additionally, it matches well with similarly shaped and sized wooden structures observed in the study of Hafeez et al., 2019 [35]. This suggests that in addition to repeated measurements, future observation-based values in regular and irregular structures could also be validated against existing measurements in published compilations, like the instrumented buildings in the CESMD (Center for Engineering Strong Motion Data) database, and targeted campaigns, such as described by Hafeez et al. [35] in their study of 41 wood-framed structures in Canada.

Furthermore, the relative consistency of the three measurements, despite differences in source and path effects of the input motion, validates the accuracy of phone-based measurements. In addition to any residual contributions from the different input ground motions, the observed 7% decrease in the frequency over four months (from 4.85 to 4.5 Hz) at low levels of shaking can be due to several reasons, including material cracking, disengagement of nonstructural elements, such as partition walls that can provide minor contributions to stiffness, foundation rocking, etc. Temperature can also affect a building’s response [38]. However, because temperatures were largely stable in this building’s area (in the San Francisco region) over the sampled time period, we do not expect a strong temperature influence in this case [39]. The observed level of change in the natural frequencies under low levels of shaking has also been documented in buildings instrumented with conventional sensors (e.g., the California Strong Motion Instrumentation Program, CSMIP).

Kohler et al., 2005 [40] monitored a well-instrumented 17-story steel-frame building in Los Angeles and observed that forced vibration measurements resulted in lower fundamental frequency results than ambient noise vibrations, even when the forced vibrations were small. This is attributed to soil–structure softening interactions, which resolved elastically in the hours after the forcing (wind or earthquake) was over. This suggests that the measurements made with MyShake, which are labeled as the response of a structure at full stiffness, may be underestimating the modal frequency as compared to the frequency in ambient conditions. However, because the MyShake catalog will be forced-vibration-based, the bias will be systematic unless the phone noise drops so low that we can begin incorporating ambient observations. Furthermore, as the small-motion deviations are elastic, MyShake’s observations are still appropriate to use as a ‘healthy’ benchmark against which observations made after potentially damaging shaking can be compared. The deviations between fully ambient and light-force motion observations are small, on the order of 0.01–0.1 Hz in the study of Kohler et al. [40], in comparison to much larger changes in the natural frequency that would be indicative of structural damage.

## 5. Conclusions

The ultimate goal of the MyShake SHM project is to increase the number of instrumented buildings significantly by making use of smartphone sensors already present in almost all buildings. We begin here with a proof-of-concept study to demonstrate the feasibility and reliability of using citizen science smartphone seismic data for this purpose. Taking the characteristics and current limitations of our smartphone network into account, we determine fundamental frequency to be the appropriate entry point into SHM. Due to the higher noise floor inherent to phones, we established that a small impulse would be required to excite a structure’s fundamental frequency enough to be measured. We therefore make measurements while buildings are vibrated by a shake table and in the coda of low-intensity earthquake shaking (when damage is not expected).

In a controlled shake table test, we compared the results recorded by phones running MyShake and traditional high-quality MEMS accelerometers and found good agreement between them, suggesting that smartphones are suitable for making these observations. In a set of seven real-world buildings for which MyShake collected a waveform, the extracted modal frequency fell within 40% of the model-based estimate for the structure based on ASCE standards (Table 4). These modeled estimates were necessarily simple due to the limited structural information known about the buildings, and perfect matches were not expected. Instead, we use this comparison to confirm our measurements are reasonable. Over time, an accumulation of measurements for a given building can be used to establish a more robust baseline for its structural health. As an example of this, we present one five-story structure for which three measurements were made in the coda of three earthquakes over the course of four months (Figure 17). The modal frequency measured in each varies within 7% of the first, decreasing from 4.85 to 4.5 Hz over time. All fit fairly well with a similarly shaped structure assessed using traditional methods by Hafeez et al., 2019 [35]. This example begins to set expectations for the accuracy of our measurements, inclusive of the slight structural softening that can sometimes accompany even low-intensity shaking.

Over time, an accumulation of ‘healthy baseline’ response information will assist in setting reasonable thresholds for how much a measurement must deviate from baseline before we should consider that a smartphone modal frequency observation indicates structural damage. Traditional structural health monitoring, based on fixed high-quality sensors, looks for the natural period of a building to permanently change by a factor of at least 1.5 times to correspond with significant structural weakening [41,42,43,44,45]. Based on the preliminary observations presented here, MyShake smartphones should be able to observe a change of this magnitude if there is a baseline observation on the natural period of a building before a quake, and then a second observation after the quake or from the coda of the quake. Furthermore, an extended aftershock sequence could be used to study possible long-term nonlinear elasticity, such as from slow-recovering soil–structure dynamics [46].

Beyond the main contributions of this project, there are still limitations, such as the use of one sensor at each building without knowing its exact elevation, and the lower sampling rate and higher noise floor of MyShake sensors compared to conventional sensors. Future studies will be planned to overcome some of these limitations, while others will be handled by the inherent advances in related technologies.

In the long term, we envision developing a database of structural information for buildings, based on ‘healthy’ behavior observed during smaller vibrations. When a severe event occurs, MyShake observations from the tail of the earthquake or successive aftershocks could be used to compute comparative values for the same structures. This rapidly available, remote diagnostic could then be distributed to local engineering first responders to assist in prioritizing inspections of buildings to those with suspected damage. As the app continues to proliferate (motivated along the US West Coast by earthquake early warning adoption) and smartphone technology continues to improve, we also envision being able to improve on our current limitations in future research. This could include recording a building’s response on multiple devices at once and resolving better where they are located within that building, which could in turn enable the resolution of additional structural health parameters. Structural health monitoring and emergency response are life-critical, but resource-limited endeavors. We are hopeful that the MyShake SHM project can contribute to a solution by providing low-cost, low-maintenance remote sensing observations.

## Figures and Tables

**Table 1:** First three mode frequencies identified using MyShake and conventional accelerometer measurements at the top of a three-story test structure tested on a shake table.

		Pre-Event		Co-Event		Post-Event	
		**MyShake**	**TE 3022**	**MyShake**	**TE 3022**	**MyShake**	**TE 3022**
**Event**	**Mode No.**	**Hz (s)**	**Hz (s)**	**Hz (s)**	**Hz (s)**	**Hz (s)**	**Hz (s)**
	1	3.52	3.52	3.32	3.32	3.50	3.47
		(0.284)	(0.284)	(0.301)	(0.301)	(0.286)	(0.288)
Kocaeli	2	10.85	10.70	10.30	10.30	10.70	10.65
Run 1		(0.0933)	(0.0920)	(0.0969)	(0.0971)	(0.0930)	(0.0939)
	3	18.14	17.67	17.64	17.57	18.12	18.04
		(0.0551)	(0.0566)	(0.0567)	(0.0569)	(0.0552)	(0.0454)
	1	3.52	3.52	2.77	3.45	3.50	3.32
		(0.284)	(0.284)	(0.361)	(0.290)	(0.286)	(0.301)
Kocaeli	2	10.70	10.87	9.50	10.52	10.72	10.67
Run 2		(0.0933)	(0.0920)	(0.1050)	(0.0951)	(0.0929)	(0.0937)
	3	18.09	18.72	17.62	17.84	17.94	18.14
		(0.0553)	(0.05340	(0.0567)	(0.0561)	(0.0557)	(0.0551)
	1	3.5	3.5	2.7	2.7	3.45	3.5
		(0.286)	(0.286)	(0.370)	(0.370)	(0.290)	(0.286)
Kocaeli	2	10.75	10.75	9.7	9.65	10.52	10.55
Run 3		(0.0930)	(0.0930)	(0.1031)	(0.1036)	(0.0951)	(0.0948)
	3	17.82	17.59	17.05	15.02	17.85	18.39
		(0.0561)	(0.0569)	(0.0587)	(0.0666)	(0.0560)	(0.0544)
	1	3.47	3.45	3.20	3.20	3.45	3.47
		(0.288)	(0.290)	(0.313)	(0.313)	(0.290)	(0.288)
Kobe	2	10.97	9.07	9.37	10.10	10.80	9.02
Run 1		(0.0912)	(0.1103)	(0.1067)	(0.0990)	(0.0926)	(0.1109)
	3	17.72	17.79	15.94	17.27	18.19	18.29
		(0.0564)	(0.0562)	(0.0627)	(0.0579)	(0.0550)	(0.0547)
	1	3.47	3.5	2.85	2.82	3.42	3.45
		(0.288)	(0.286)	(0.351)	(0.355)	(0.292)	(0.290)
Kobe	2	10.77	9.02	9.17	9.2	10.82	9
Run 2		(0.0929)	(0.1109)	(0.1091)	(0.1087)	(0.0924)	(0.1111)
	3	17.82	18.67	16.29	16.29	17.97	17.97
		(0.0561)	(0.0536)	(0.0614)	(0.0614)	(0.0556)	(0.556)

**Table 2:** A summary of analyzed structures for which MyShake phones recorded a waveform during earthquake shaking and the identified modal frequencies. Results are ordered in the sequence that their corresponding figures appear in the paper.

	Event Magnitude,		Identified	Identified	Potential
**Building**	**Location, and**	**Epicentral**	**Natural**	**Natural**	**Second**
**No. Stories, Use Type**	**UTC Datetime**	**Distance**	**Frequency**	**Period**	**Mode**
8-story hotel	Mw 4.4	16 km	2.08 Hz	0.481 s	
	Berkeley, CA, USA				6.57 Hz
	4 January 2018 10:39:37				(0.152 s)
14-story condo complex	Mw 4.28	24 km	1.42 Hz	0.704 s	
	Carson, CA, USA				9.91 Hz
	18 September 2021 02:58:34				(0.101 s)
18-story apartment building	Mw 5.37	212 km	0.82 Hz	1.22 s	
	Searles Valley, CA, USA				2.9 Hz
	5 July 2019 11:07:53				(0.345 s)
3-story apartment building	Mw 4.28	25 km	2.71 Hz	0.339 s	
	Carson, CA, USA				8.95 Hz
	18 September 2021 02:58:34				(0.112 s)
5-story apartment building	Mwr 4.7	73 km	3.05 Hz	0.328 s	
	Tsukuba, Japan				—
	7 September 2016 04:28:49				
3-story hotel	Mw 6.4	117 km	4.26 Hz	0.235 s	
	Ferndale, CA, USA				—
	20 December 2022 10:34:24				
5-story apartment complex	Ml 4.29	80 km	4.85 Hz	0.206 s	
	Santa Rosa, CA, USA				11.54 Hz
	14 September 2022 01:40:20				(0.0867 s)
	Mw 5.06	85 km	4.62 Hz	0.216 s	
	Alum Rock, CA, USA				13.33 Hz
	25 October 2022 18:42:02				(0.0750 s)
	Mw 3.57	17 km	4.50 Hz	0.222 s	
	El Cerrito, CA, USA				11.04 Hz
	17 December 2022 11:39:42				(0.0910 s)

**Table 3:** Variables for use in Equation (1).

Structural System Type	$C_{t}$	*x*
Steel moment-resisting frame	0.028	0.8
Reinforced concrete moment-resisting frame	0.016	0.9
Eccentrically or buckling-restrained braced frame	0.030	0.75
Concrete shear wall	0.020	0.75
Wood buildings	0.032	0.55
Other	0.020	0.75

**Table 4:** A comparison of the observed natural frequencies compared to a range of model-based options. Where appropriate, we use Equations (1) and (2) to compute expected values. Since framing materials are not known for certain for most of the buildings we observe, we provide a range of likely values. The ‘steel’ category uses the coefficient for moment-resisting frames. Other types of steel construction would be associated with a natural period in between that of moment-resisting concrete and the ‘other’ category. Concrete shear wall construction uses the same values as ‘other’. Building heights were sourced from OSM Buildings and converted to feet for use in the ASCE standards.

			10/n		$T_{a}=C_{t}h^{x}$	Observation
	**Height**	**Observed**	**Frequency**		**Frequency**	**Difference**
	**from OSM**	**Frequency**	**Estimate**	**Framing**	**Estimate**	**from Equation (1)**
**Structure**	**Buildings**	**(Period)**	**(Period)**	**Types**	**(Period)**	**Estimate**
			1.25 Hz	Concrete	0.948 Hz	+119%
			(0.8 s)		(1.055 s)	
8-story hotel	32 m	2.08 Hz		Other	1.524 Hz	+36%
		(0.481 s)			(0.656 s)	
				Wood	2.417 Hz	−14%
					(0.414 s)	
				Steel	0.655 Hz	+117%
					(1.528 s)	
14-story	45.2 m	1.42 Hz	n/a	Concrete	0.695 Hz	+104%
condo complex		(0.704 s)			(1.439 s)	
				Other	1.177 Hz	+21%
					(0.85 s)	
				Steel	0.591 Hz	+39%
					(1.691 s)	
18-story	51.3 m	0.82 Hz	n/a	Concrete	0.62 Hz	+32%
apartment building		(1.22 s)			(1.613 s)	
				Other	1.07 Hz	−23%
					(0.935 s)	
			3.33 Hz	Concrete	2.021 Hz	+34%
			(0.3 s)		(0.495 s)	
3-story	13.8 m	2.71 Hz		Other	2.865 Hz	−5%
apartment building		(0.369 s)			(0.349 s)	
				Wood	3.838 Hz	−29%
					(0.261 s)	
			2.0 Hz	Concrete	1.516 Hz	+101%
5-story	19 m	3.05 Hz	(0.5 s)		(0.66 s)	
apartment building		(0.328 s)		Other	2.254 Hz	+35%
					(0.444 s)	
3-story hotel	12 m	4.26 Hz	n/a	Wood	4.145	+3%
		(0.235 s)			(0.241 s)	
		4.85 Hz				
		(0.206 s)	2.0 Hz	Concrete	1.684 Hz	+167 to 188%
			(0.5 s)		(0.594 s)	
5-story	16.9 m	4.62 Hz		Other	2.461 Hz	+83 to 97%
apartment complex		(0.216 s)			(0.406 s)	
				Wood	3.433 Hz	+31 to 41%
		4.5 Hz			(0.291 s)	
		(0.222 s)				

## Data Availability

The waveform data presented in this study are not publicly available due to privacy concerns for MyShake app users, including protection of sensitive GPS location information. MyShake’s privacy policy restricts most data availability to members of the project only. Waveforms with all sensitive metadata removed may be available on request.

## Abbreviations

The following abbreviations are used in this manuscript:

MDPI	Multidisciplinary Digital Publishing Institute
DOAJ	Directory of Open Access KJurnals
SHM	Structural Health Monitoring
MEMs	Micro-electromechanical Systems
BSDN	Berkeley Digital Seismic Network
dB	Decibels
NTP	Network Time Protocol
GPS	Global Positioning System
PEER	Pacific Earthquake Engineering Research Center
SMA	Shape Memory Alloy
DOF	Degree of Freedom
PSD	Power Spectral Density
Mw	Moment Magnitude
Mwr	Regional Moment Magnitude
Ml	Local Magnitude (also known as Richter Magnitude)
ASCE	American Society for Civil Engineers
CESMD	Center for Engineering Strong Motion Data
CSMIP	California Strong Motion Instrumentation Program
